# Fermented *Clostridium butyricum* GKB7 Inhibits Osteoarthritis Induced by Anterior Cruciate Ligament Transection in a Preclinical Model

**DOI:** 10.7150/ijms.126850

**Published:** 2026-02-18

**Authors:** Wei-Cheng Chen, Li-Chai Chen, You-Shan Tsai, Yen-Po Chen, Yen-Lien Chen, Chin-Chu Chen, Yen-You Lin, Tzu-Ching Chang, Chen-Ming Su, Chih-Hsin Tang

**Affiliations:** 1 Department of Medicine, MacKay Medical University, New Taipei, Taiwan.; 2 Division of Sports Medicine & Surgery, Department of Orthopedic Surgery, MacKay Memorial Hospital, Taipei, Taiwan.; 3 Department of Pharmacy, Tajen University, Pingtung, Taiwan.; 4 Biotech Research Institute, Grape King Bio Ltd., Taoyuan City, Taiwan.; 5 Institute of Food Science and Technology, National Taiwan University, Taipei City, Taiwan.; 6 Translational Medicine Center, Shin Kong Wu Ho-Su Memorial Hospital, Taipei, Taiwan.; 7 Department of Pharmacology, School of Medicine, China Medical University, Taichung, Taiwan.; 8 Department of Sports Medicine, College of Health Care, China Medical University, Taichung, Taiwan.; 9 Chinese Medicine Research Center, China Medical University, Taichung, Taiwan.; 10 Department of Medical Laboratory Science and Biotechnology, College of Medical and Health Science, Asia University, Taichung, Taiwan.

**Keywords:** osteoarthritis, fermented GKB7, probiotics

## Abstract

Aging-related osteoarthritis (OA) affects joints and causes functional impairment. Probiotics are recognized safe for consumption and numerous exhibit beneficial bioactivity for human health conditions. *Clostridium butyricum*, frequently found in the environment and reported to colonize the stomachs of adults and newborns, exhibits anti-inflammatory properties. This study investigated whether fermented* Clostridium butyricum* GKB7 is effective at preventing the advancement of OA. Fermented GKB7 reduces bone pain and the development of OA associated with anterior cruciate ligament transaction. Through the reduction of pro-inflammatory cytokines IL-1β and TNF-α, as well as the chondrolytic factors MMP-3, MMP-13, and ADAMTS5, fermented GKB7 inhibited the degradation of aggrecan and COL2A1. Bone loss and cartilage degradation were blocked as a result of this activity. According to our findings, fermented GKB7 enhances the prevention of OA development.

## Introduction

Osteoarthritis (OA) is one of the most prevalent degenerative conditions, which have become some of the most frequent health problems due to medical advancements and increased life expectancy. According to the Global Burden of Disease study, OA affected over 528 million people worldwide, and over a ten-year period, its prevalence increased 1, 2. Pathological characteristics of OA include inflammation of the synovial region, cartilage deterioration, and subchondral bone sclerosis. These characteristics cause joint discomfort and stiffness and are frequently irreversible at the time of diagnosis 3, 4. There is currently no known cure for OA, and the only available treatments are to reduce discomfort or halt the disease's progression.

Joint discomfort, structural damage, and the release of synovial fluid—all of which are critical in fostering inflammation and tissue deterioration in OA—are closely associated with chronic inflammation of the synovial tissues 5-7. Proinflammatory cytokines such as TNF-α and IL-1β, as well as synovium-related factors including MMP-3, MMP-13, and ADAMTS5, are significantly correlated with the severity of knee OA and may be associated with its progression 8-11. Aggrecan and collagen II are among the components of the cartilage matrix that break down as a result of increased levels of inflammatory mediators and degradative agents 12, 13. Relevant research suggests that strategies that reduce inflammation may be used to treat OA 5, 14.

Because probiotics are deemed harmless for consumption and many have beneficial functional activity for human ailments, they have become popular targets for research and development in the treatment of OA 15. *Clostridium butyricum*, an anaerobic bacillus recognized for its production of butyric acid, is commonly found in the environment and has been documented to colonize about 10-20% of the stomachs of both adults and newborns 16. Non-pathogenic strains of *Clostridium butyricum* are utilized as probiotic supplements for the treatment or prevention of gastrointestinal infections and various other conditions, such as metabolic diseases, bowel disease, multiple sclerosis, and neurodegenerative diseases 16-19. In our earlier work, we documented that *Clostridium butyricum* GKB7 mitigates the progression of OA by blocking inflammatory cytokine generation associated with it 20. Both live and dead GKB7 exhibit anti-inflammatory functions 21. This study investigated whether fermented GKB7 is effective at preventing the advancement of OA. In this study, we discovered that fermented GKB7 prevent the onset of OA triggered by anterior cruciate ligament transection (ACLT)* in vivo*.

## Materials and Methods

### Materials

Abcam (Cambridge, UK) provided the aggrecan (ab3778) antibody. Santa Cruz Biotechnology (Dallas, TX, USA) provided the MMP-3 (SC-21732) and MMP-13 (SC-30073) antibodies. We purchased TNF-α (A11534), COL2A1 (A1560), and ADAMTS5 (A2836) antibodies from ABclonal, Inc. (Woburn, MA, USA). R&D Systems, Inc. (Minneapolis, MN, USA) provided the IL-1β (MAB601) antibody.

### Preparation of fermented GKB7

The *Clostridium butyricum* GKB7 strain was obtained from fecal samples of healthy individuals in Taiwan, as detailed in our earlier report 20. The fermented GKB7 was prepared according to our previous report 22.

### ACLT animal model

We purchased eight-week-old male Sprague Dawley (SD) rats weighing between 300 and 350 g from the National Laboratory Animal Center in Taipei, Taiwan. They were divided into three groups at random: ACLT alone, ACLT plus fermented GKB7 (100 mg/kg), and sham surgery (controls). The process outlined in our previous documents was followed for performing the ACLT operations 23, 24.

The weight-bearing incapacitance test was examined weekly to evaluate spontaneous discomfort after ACLT, based on differences in dynamic weight bearing between the resting right and left hind limbs, in accordance with our previous protocols 25, 26.

### μ-CT measurements

The rats were sacrificed after six weeks of treatment. Following our previous techniques, their undamaged right knee joints were scanned using a SkyScan 2211 μ-CT scanner (Bruker; Kontich, Belgium) and processed using CTAn software 25, 27.

### Histological analysis

Hematoxylin and eosin (H&E) and Safranin-O/Fast Green stains were used to investigate histological alterations in OA tissue under an optical microscope, as previously reported 28, 29. Tissues from knee joints were decalcified using 10% EDTA after being fixed in 4% formaldehyde. Ethanol dehydration came next. After being embedded in paraffin blocks, the specimens were cut into sections that were 5 µm thick for histological staining. The structural alterations in the cartilage of the central weight-bearing region of the medial tibial plateau were assessed using the Osteoarthritis Research Society International (OARSI) histopathological assessment system 30, 31. This system uses staging and grading scores to show the severity of OA and the depth of lesions, respectively.

### Immunohistochemistry (IHC) staining

The Leica Novolink Polymer Detection system (Leica Biosystems Inc., IL, USA) was used for the immunohistochemical examination, as described in reference 32, 33. After a brief application of 3% hydrogen peroxide, tissue slices were treated with 3% BSA. After applying primary antibodies to the sections, they were stained with diaminobenzidine substrate and incubated with a secondary antibody coupled with peroxidase for an hour.

### Statistical analysis

Statistical analyses for quantified results were conducted using GraphPad Prism 5.0 software. Data are presented as the mean ± standard deviation (S.D.). The paired sample t-test and One-way ANOVA followed by Bonferroni post hoc testing was used to compare results from two groups and from more than two groups, respectively. Statistical significance was determined by a *p*-value of less than 0.05 in all cases.

## Results

### Fermented GKB7 do not affect the body weight growth curve

**Figure 1:**
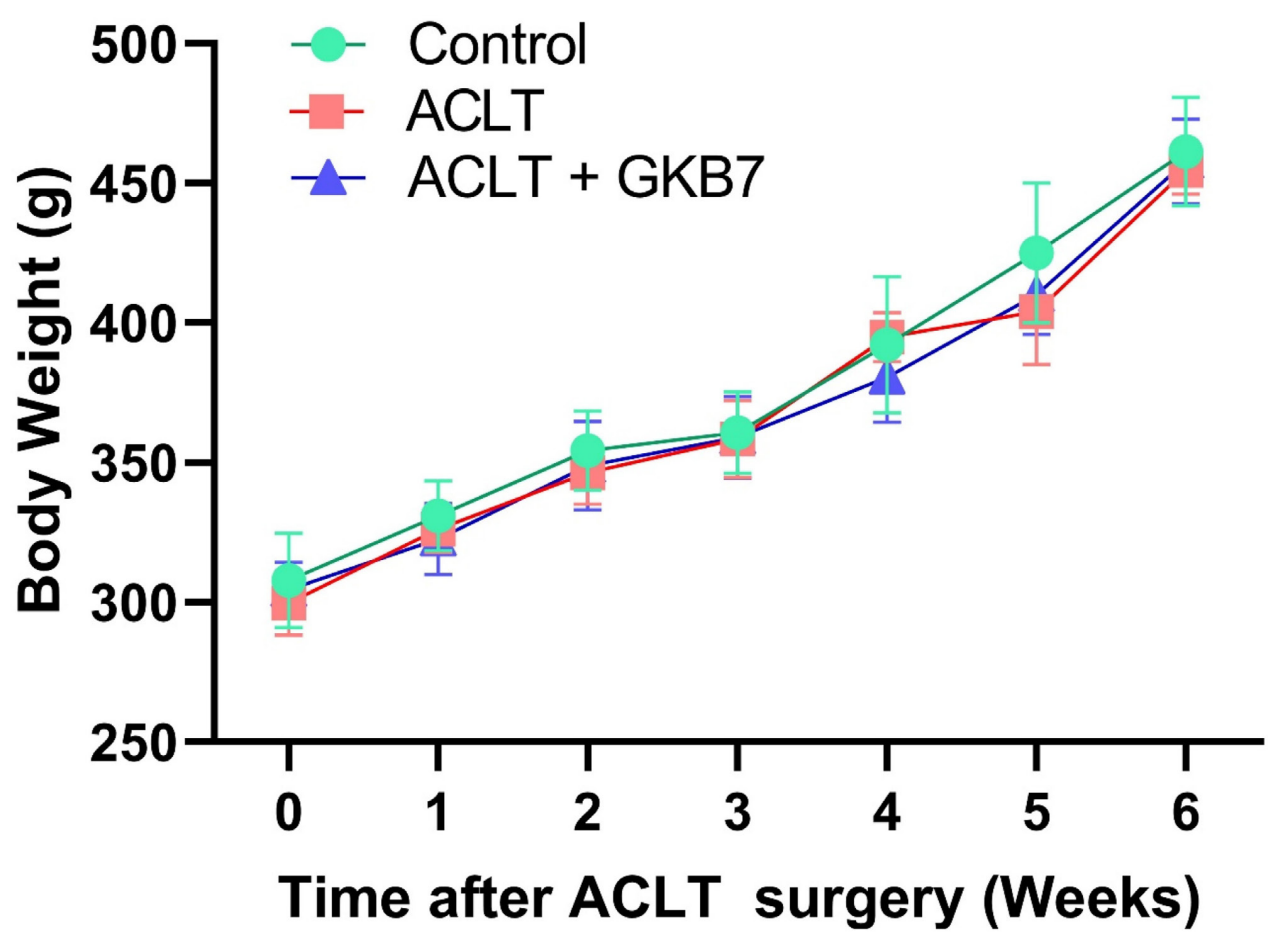
** Increase in body weight throughout the experimental phase.** Throughout the course of the experiment, body weight was measured.

We investigated the preventive benefits of fermented GKB7 using a rat model of ACLT-induced knee arthritis. To look into the underlying mechanisms, assessments of pain behavior and histological investigations were performed. The rats' body weights were recorded the day before surgery, and this procedure was repeated every week until the rodents were put to death. All groups gradually increased in body weight during the course of the trial, and there were no discernible differences between the groups (Figure 1). According to our research, fermented GKB7 has no harmful negative effects on body weight.

### Fermented GKB7 diminishes OA pain

**Figure 2:**
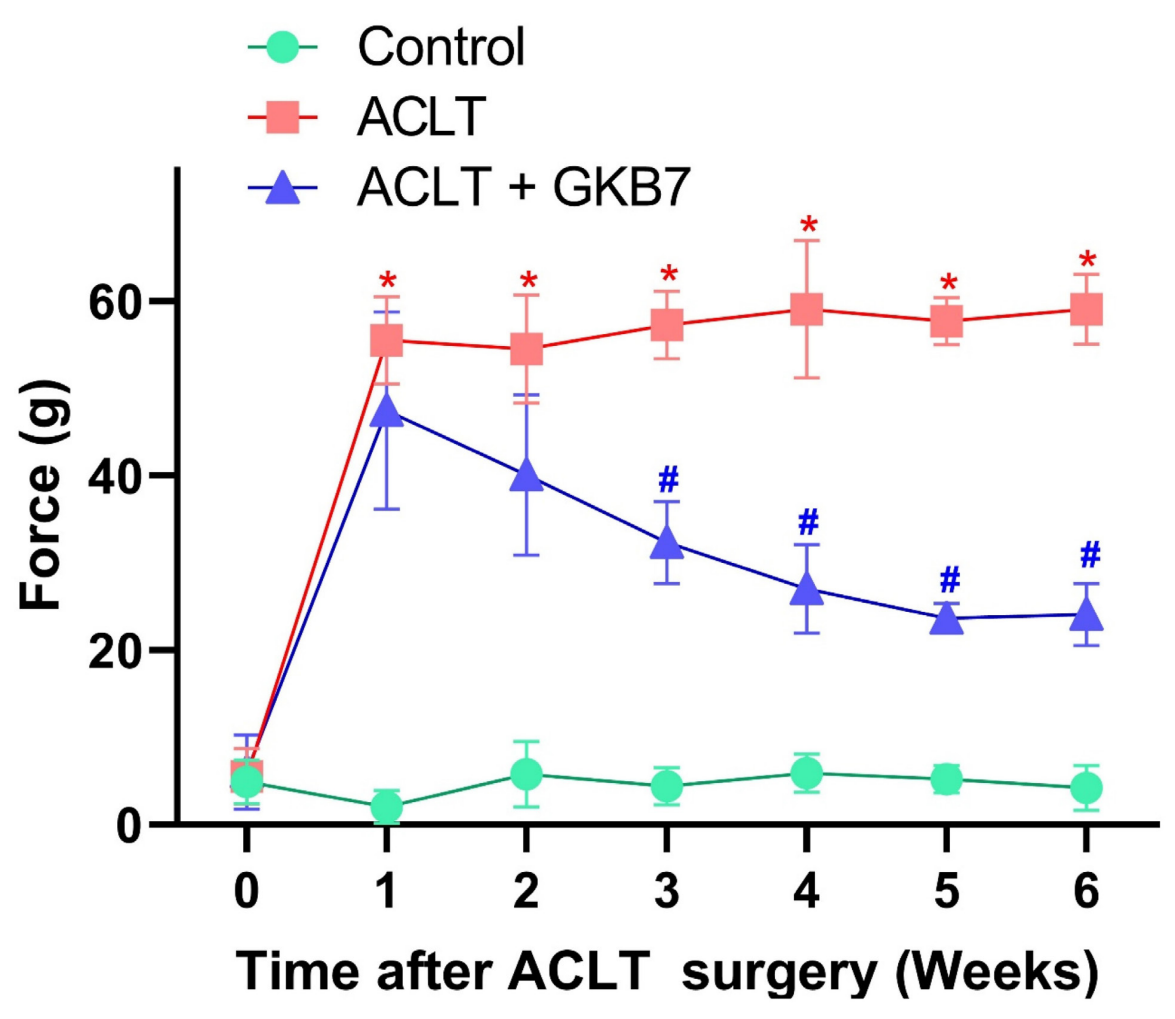
** Fermented GKB7 decelerates ACLT-induced bone pain.** Every week, weight-bearing behavioral testing was conducted to assess deficits in weight-bearing forces. * *p*<0.05 compared with the control group; # *p*<0.05 compared with the ACLT-only group.

The rats' pain behavior was examined using the static weight-bearing incapacitance test. All groups showed a significant unequal weight-bearing posture during the first week following surgery (Figure 2). Throughout the experiment, this significant imbalance became more pronounced in the ACLT animals. However, there were notable improvements in pain-related behavior in the ACLT+ fermented GKB7 group (Figure 2). These findings imply that fermented GKB7 successfully reduces pain associated with OA.

### Fermented GKB7 protects against ACLT-triggered osseous and cartilage damage in an ACLT-triggered OA model

**Figure 3:**
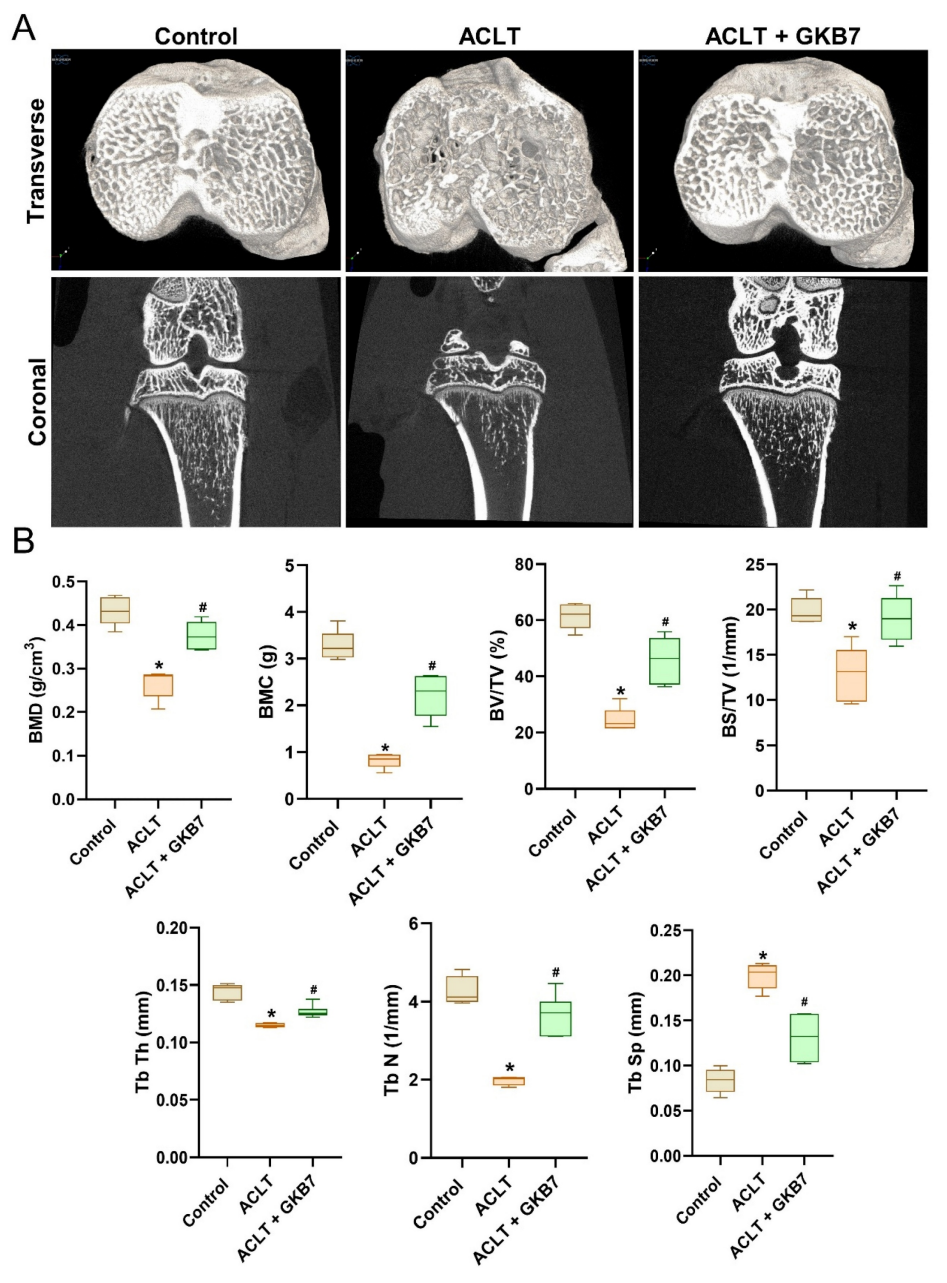
** Fermented GKB7 ameliorates osseous damage in the ACLT-induced OA knee joint.** (A) Representative micro-CT images from knee subchondral bone. (B) Quantitative analyses of BMD, BMC, BV/TV, BS/TV, Tb.Th, Tb.N, and Tb.Sp. * *p*<0.05 compared with the control group; # *p*<0.05 compared with the ACLT-only group.

Changes in trabecular microarchitecture were assessed by μ-CT six weeks following ACLT surgery. Significant bone deterioration was seen in ACLT rats as compared to controls, demonstrating the OA lesion brought on by ACLT surgery (Figure 3). Bone mineral density (BMD), bone mineral content (BMC), bone volume/tissue volume ratio (BV/TV), bone surface to tissue volume ratio (BS/TV), trabecular thickness (Tb.Th), trabecular number (Tb.N), and trabecular separation (Tb.Sp) all decreased in ACLT rats, according to quantitative evaluation (Figure 3). Additionally, rats treated with fermented GKB7 showed significant improvements in bone microstructure when compared to the ACLT group (Figure 3).

**Figure 4:**
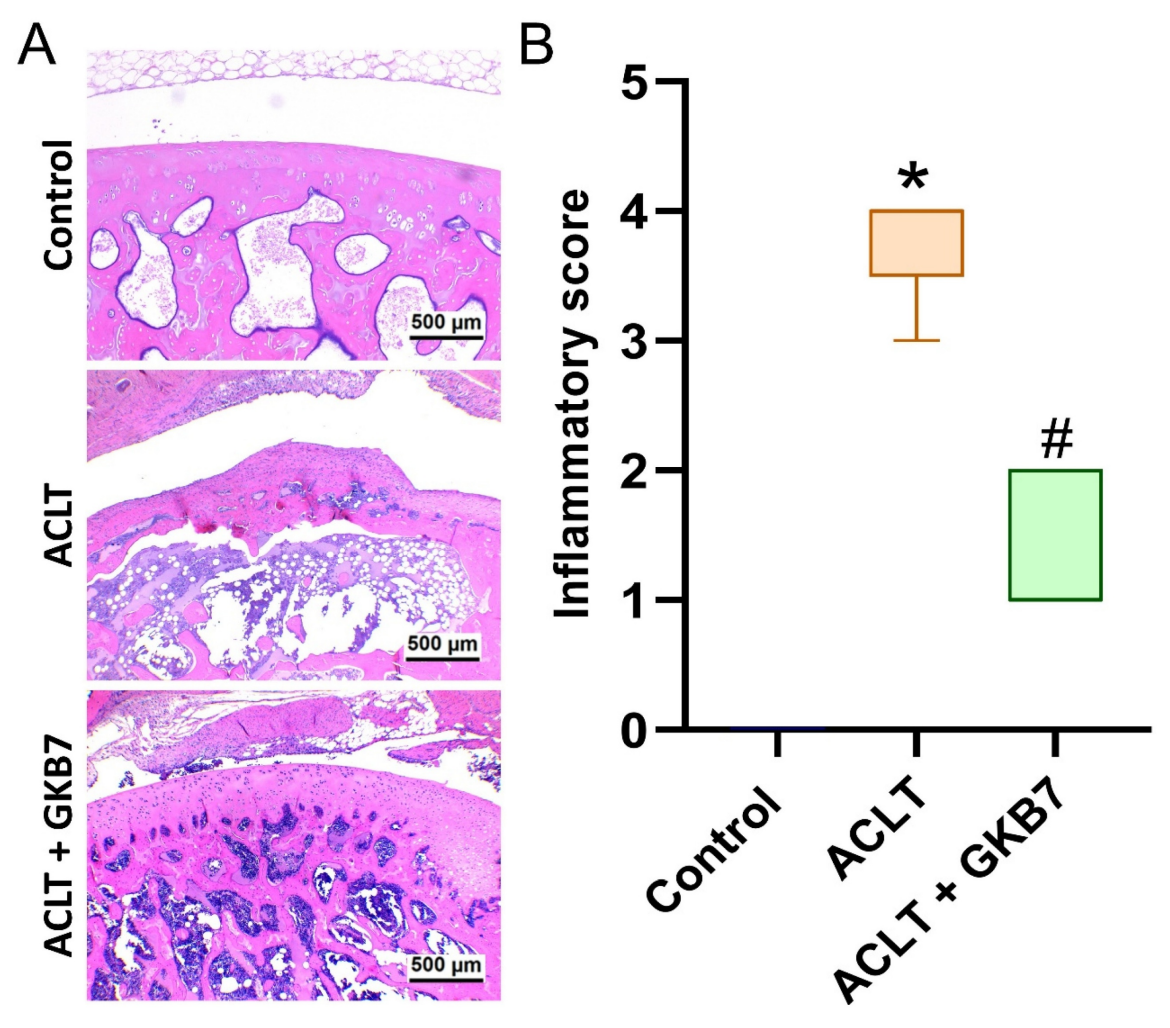
** Fermented GKB7 blocks ACLT-induced synovial inflammation and cartilage degradation.** (A) Histological sections from knees stained with H&E. (B) Quantitative analyses of synovium scores. Scale bar = 500 μm. * *p*<0.05 compared with the control group; # *p*<0.05 compared with the ACLT-only group.

**Figure 5:**
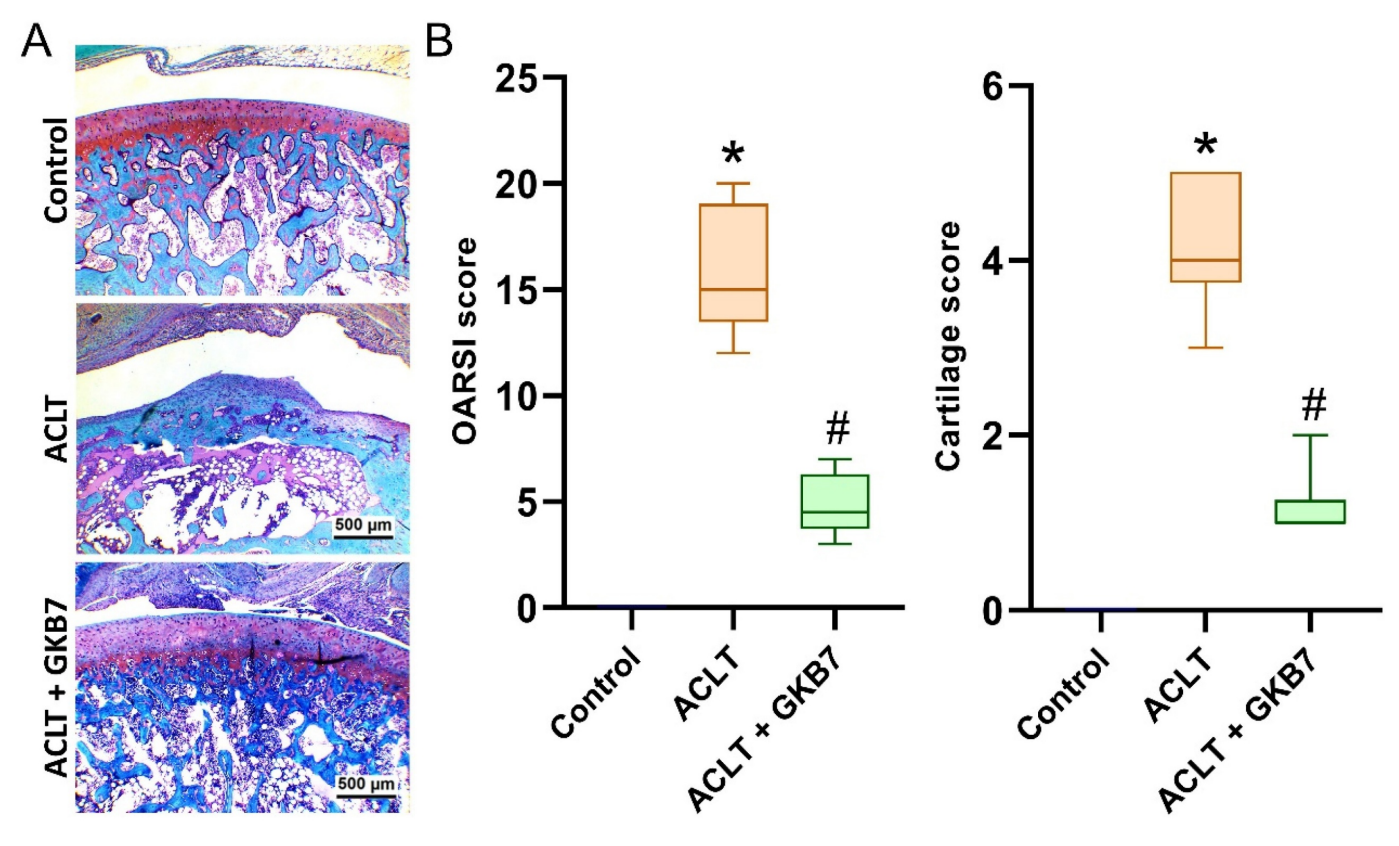
** Fermented GKB7 blocks ACLT-induced cartilage breakdown.** (A) Histological sections from knees stained with Safranin-O. (B) Quantitative analyses of OARSI and cartilage scores. Scale bar = 500 μm. * *p*<0.05 compared with the control group; # *p*<0.05 compared with the ACLT-only group.

In the ACLT knee groups, histological evaluation using H&E and Safranin-O/Fast Green staining revealed articular cartilage degeneration and synovial lining hyperplasia (Figure 4 and 5). The ACLT+fermented GKB7 group had less pathological changes in cartilage tissue and less synovial tissue hyperplasia than the ACLT group, according to the quantification of inflammation, OARSI scores, and cartilage scores (Figure 4 and 5).

### Fermented GKB7 suppress proinflammatory cytokine production and cartilage degradation

**Figure 6:**
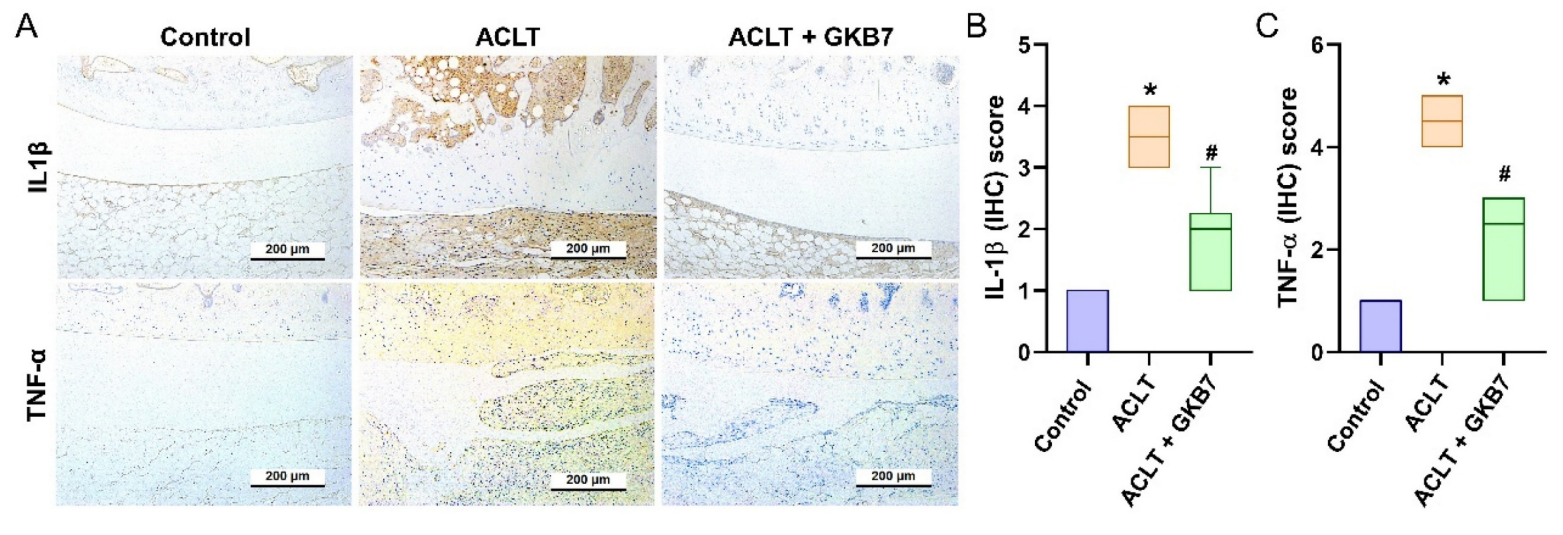
** Fermented GKB7 diminishes the induction of IL-1β and TNF-α in ACLT-induced OA articular cartilage.** Immuno-histochemistry analysis and scoring of IL-1β (A, B) and TNF-α (A, C) in rat knee joint cartilage. Scale bar = 200 μm. * *p*<0.05 compared with the control group; # *p*<0.05 compared with the ACLT-only group.

**Figure 7:**
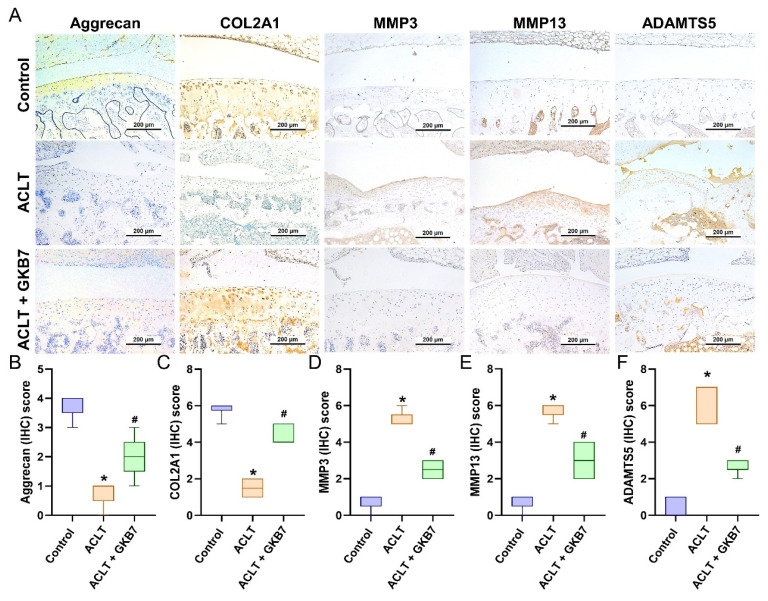
** Fermented GKB7 reserves the expression of aggrecan and COL2A1 accompanying with suppression of MMP-3, MMP-13 and ADAMTS5 in ACLT-induced OA articular cartilage.** (A) Immuno-histochemistry analysis MMP3, MMP-13, ADAMTS5, aggrecan and COL2A1 in rat knee joint cartilage. (B-F) Scoring of the immunosignals of MMP3, MMP-13, ADAMTS5, aggrecan and COL2A1. Scale bar = 200 μm. * *p*<0.05 compared with the control group; # *p*<0.05 compared with the ACLT-only group.

The IHC analysis revealed a significant increase in TNF-α and IL-1β production in the ACLT group's synovial tissue, indicating an increase in inflammatory activity. This rise was considerably reduced in the ACLT+fermented GKB7 group, as shown in Figure 6. Further evaluation of cartilage metabolism was carried out using IHC labeling of MMP-3, MMP-13, ADAMTS5, aggrecan, and type II collagen alpha1 chain (COL2A1), which is the foundation for articular cartilage. The ACLT+fermented GKB7 group showed higher levels of aggrecan and COL2A1 and lower levels of MMP-3, MMP-13, and ADAMTS5 in comparison to the ACLT group (Figure 7).

## Discussion

A variety of probiotic strains applied as supplements have demonstrated therapeutic functions for human ailments, including arthritis, respiratory and gastrointestinal issues 34, 35. Both preclinical and clinical reports reveal that probiotics may alleviate OA-related pain in individuals by positively modulating gut microbiota and reducing inflammation through different mechanisms for the treatment of OA 36. Furthermore, preclinical trials indicate that probiotics can hinder cartilage breakdown and the advancement of OA models 37. *Lactobacillus plantarum* is said to reduce the rise in bone erosion and cartilage degradation induced by ACLT 15. Combination of the probiotic strains *Lactiplantibacillus plantarum* and *Lacticaseibacillus paracasei* notably reduced cartilage erosion at the medial femoral condyle in a mouse model with medical meniscus destabilization 37. Probiotics such as *Clostridium butyricum* have been employed to treat or avert a range of conditions, including necrotic enteritis, diabetes, ulcerative colitis, bowel disorder, liver injury, dementia, pouchitis, and antibiotic-induced diarrhea 38, 39. We previously found that live and dead GKB7 exhibits anti-inflammatory and chondroprotective functions in an OA model 21. Here, our investigation further revealed that fermented GKB7 reduces OA-related pain and progression in an ACLT-promoted OA model. Fermented GKB7 inhibits inflammatory cytokine expression and cartilage degradation *in vivo*. Fermented GKB7 also serves as a potential supplement for OA management.

OA is a chronic inflammatory disease that affects cartilage degradation, synovial inflammation, and pain behavior 2, 40. Inflammatory cytokines, such as TNF-α and IL-1β, play a major role in the progression of OA, causing joint pain, increased inflammatory reactions, and abnormalities in chondrocyte metabolism 14, 41. Additionally, clinical data from previous investigations showed that OA patients have markedly higher serum and synovial tissue levels of TNF-α and IL-1β 23, 42. IL-1β and TNF-α are important targets for finding successful OA treatment approaches during pre-clinical trials. Our ACLT-induced OA model demonstrated that ACLT surgery replicates clinical features, resulting in increased TNF-α and IL-1β production in synovial tissue and cartilage. The administration of fermented GKB7 clearly resulted in a downregulation of TNF-α and IL-1β production in synovial and cartilage tissues, suggesting that fermented GKB7's anti-OA properties stem from its ability to prevent the synthesis of TNF-α and IL-1β.

Collagen and the proteoglycan aggrecan make up the majority of cartilage's gel-like matrix. Proteoglycans draw water portions to form a gel that preserves the cartilage's robust and inflated properties, while COL2, the main component of the matrix, builds a fibrous network foundation 43. Chondrocytes are supported in maintaining stability and a balanced metabolism by the cartilage matrix 44. When chondrocytes are unable to maintain metabolic homeostasis within the cartilage matrix, cartilage-related diseases like joint inflammation and articular degradation arise 45, 46. Here, we discovered that ACLT increased the synthesis of the chondrolytic factors MMP-3, MMP-13, and ADAMTS5 while decreasing the expression of aggrecan and COL2A1. By reducing the expression of chondrolytic factors, fermented GKB7 restores chondroprotective qualities and slows the progression of OA.

The limitations of the current study should be acknowledged. Although we provided evidence that fermented GKB7 prevents ACLT-induced OA progression, we did not elucidate the underlying mechanisms of action of fermented GKB7. Future studies should investigate these mechanisms in greater detail using *in vitro* cell culture systems.

To sum up, our findings demonstrate that fermented GKB7 reduces bone pain and the development of OA related with ACLT. Through the reduction of pro-inflammatory cytokines IL-1β and TNF-α, as well as the chondrolytic factors MMP-3, MMP-13, and ADAMTS5, fermented GKB7 inhibited the degradation of aggrecan and COL2A1. This action resulted in a blockade of cartilage breakdown and bone loss. The fermented GKB7 improves the prevention of OA progression.

## References

[1] Diseases GBD, Injuries C (2020). Global burden of 369 diseases and injuries in 204 countries and territories, 1990-2019: a systematic analysis for the Global Burden of Disease Study 2019. Lancet.

[2] Allen KD, Thoma LM, Golightly YM (2022). Epidemiology of osteoarthritis. Osteoarthritis Cartilage.

[3] Hugle T, Geurts J (2017). What drives osteoarthritis?-synovial versus subchondral bone pathology. Rheumatology (Oxford).

[4] Sarhan RS, El-Hammady AM, Marei YM, Elwia SK, Ismail DM, Ahmed EAS (2025). Plasma levels of miR-21b and miR-146a can discriminate rheumatoid arthritis diagnosis and severity. BioMedicine.

[5] Kloppenburg M (2023). Inflammation is a relevant treatment target in osteoarthritis. Lancet.

[6] Chang JW, Tang CH (2024). The role of macrophage polarization in rheumatoid arthritis and osteoarthritis: Pathogenesis and therapeutic strategies. Int Immunopharmacol.

[7] Achudhan D, Liu SC, Lin YY, Huang CC, Tsai CH, Ko CY (2021). Antcin K Inhibits TNF-alpha, IL-1beta and IL-8 Expression in Synovial Fibroblasts and Ameliorates Cartilage Degradation: Implications for the Treatment of Rheumatoid Arthritis. Front Immunol.

[8] Plsikova Matejova J, Spakova T, Harvanova D, Lacko M, Filip V, Sepitka R (2021). A Preliminary Study of Combined Detection of COMP, TIMP-1, and MMP-3 in Synovial Fluid: Potential Indicators of Osteoarthritis Progression. Cartilage.

[9] Sulastri D, Arnadi A, Afriwardi A, Desmawati D, Amir A, Irawati N (2023). Risk factor of elevated matrix metalloproteinase-3 gene expression in synovial fluid in knee osteoarthritis women. PLoS One.

[10] Billesberger LM, Fisher KM, Qadri YJ, Boortz-Marx RL (2020). Procedural Treatments for Knee Osteoarthritis: A Review of Current Injectable Therapies. Pain Res Manag.

[11] Achudhan D, Li-Yun Chang S, Liu SC, Lin YY, Huang WC, Wu YC (2022). Antcin K inhibits VCAM-1-dependent monocyte adhesion in human rheumatoid arthritis synovial fibroblasts. Food Nutr Res.

[12] MᵃᶜDonald IJ, Liu SC, Huang CC, Kuo SJ, Tsai CH, Tang CH (2019). Associations between Adipokines in Arthritic Disease and Implications for Obesity. International journal of molecular sciences.

[13] MacDonald IJ, Liu SC, Su CM, Wang YH, Tsai CH, Tang CH (2018). Implications of Angiogenesis Involvement in Arthritis. International journal of molecular sciences.

[14] Hou CH, Fong YC, Tang CH (2011). HMGB-1 induces IL-6 production in human synovial fibroblasts through c-Src, Akt and NF-kappaB pathways. J Cell Physiol.

[15] Lin YY, Chang SL, Liu SC, Achudhan D, Tsai YS, Lin SW (2022). Therapeutic Effects of Live Lactobacillus plantarum GKD7 in a Rat Model of Knee Osteoarthritis. Nutrients.

[16] Stoeva MK, Garcia-So J, Justice N, Myers J, Tyagi S, Nemchek M (2021). Butyrate-producing human gut symbiont, Clostridium butyricum, and its role in health and disease. Gut Microbes.

[17] Lee J, Park SB, Kim HW, Lee HS, Jee SR, Lee JH (2022). Clinical Efficacy of Probiotic Therapy on Bowel-Related Symptoms in Patients with Ulcerative Colitis during Endoscopic Remission: An Observational Study. Gastroenterol Res Pract.

[18] Zhou M, Yuan W, Yang B, Pei W, Ma J, Feng Q (2022). Clostridium butyricum inhibits the progression of colorectal cancer and alleviates intestinal inflammation via the myeloid differentiation factor 88 (MyD88)-nuclear factor-kappa B (NF-kappaB) signaling pathway. Ann Transl Med.

[19] Chen H, Ma X, Liu Y, Ma L, Chen Z, Lin X (2019). Gut Microbiota Interventions With Clostridium butyricum and Norfloxacin Modulate Immune Response in Experimental Autoimmune Encephalomyelitis Mice. Front Immunol.

[20] Chang SL, Lin YY, Liu SC, Tsai YS, Lin SW, Chen YL (2022). Oral Administration of Clostridium butyricum GKB7 Ameliorates Signs of Osteoarthritis in Rats. Cells.

[21] Chen LC, Lin YY, Tsai YS, Chen CC, Chang TC, Chen HT (2024). Live and Dead Clostridium butyricum GKB7 Diminish Osteoarthritis Pain and Progression in Preclinical Animal Model. Environ Toxicol.

[22] Yang LC, Li TJ, Hu YF, Tsai YS, Wang CS, Lin SW (2025). Heat-inactivated Lactobacillus casei strain GKC1 Mitigates osteoporosis development in vivo via enhanced osteogenesis. Biochemical and biophysical research communications.

[23] Lee KT, Su CH, Liu SC, Chen BC, Chang JW, Tsai CH Cordycerebroside A inhibits ICAM-1-dependent M1 monocyte adhesion to osteoarthritis synovial fibroblasts. Journal of food biochemistry. 2022: e14108.

[24] Su C-H, Lin C-Y, Tsai C-H, Lee H-P, Lo L-C, Huang W-C (2021). Betulin suppresses TNF-α and IL-1β production in osteoarthritis synovial fibroblasts by inhibiting the MEK/ERK/NF-κB pathway. Journal of Functional Foods.

[25] Liu SC, Tsai CH, Wang YH, Su CM, Wu HC, Fong YC (2022). Melatonin abolished proinflammatory factor expression and antagonized osteoarthritis progression in vivo. Cell death & disease.

[26] Chen WC, Lu YC, Kuo SJ, Lin CY, Tsai CH, Liu SC (2020). Resistin enhances IL-1beta and TNF-alpha expression in human osteoarthritis synovial fibroblasts by inhibiting miR-149 expression via the MEK and ERK pathways. FASEB J.

[27] Lin YY, Ko CY, Liu SC, Wang YH, Hsu CJ, Tsai CH (2021). miR-144-3p ameliorates the progression of osteoarthritis by targeting IL-1beta: Potential therapeutic implications. J Cell Physiol.

[28] Lee HP, Chen PC, Wang SW, Fong YC, Tsai CH, Tsai FJ (2019). Plumbagin suppresses endothelial progenitor cell-related angiogenesis in vitro and in vivo. Journal of Functional Foods.

[29] Lee HP, Wang SW, Wu YC, Lin LW, Tsai FJ, Yang JS (2020). Soya-cerebroside inhibits VEGF-facilitated angiogenesis in endothelial progenitor cells. Food Agr Immunol.

[30] Hou PW, Liu SC, Tsay GJ, Chang YS, Huang HC, Tang CH (2023). High-dose Tiger-Gian formula protects the knee joint from surgically induced osteoarthritis in rats. Int J Rheum Dis.

[31] Chen WC, Lin CY, Kuo SJ, Liu SC, Lu YC, Chen YL (2020). Resistin Enhances VCAM-1 Expression and Monocyte Adhesion in Human Osteoarthritis Synovial Fibroblasts by Inhibiting MiR-381 Expression through the PKC, p38, and JNK Signaling Pathways. Cells.

[32] Achudhan D, Liu SC, Lin YY, Lee HP, Wang SW, Huang WC (2022). Antcin K inhibits VEGF-dependent angiogenesis in human rheumatoid arthritis synovial fibroblasts. Journal of food biochemistry.

[33] Wu YH, Kuo YH, Lin YY, Shieh TM, Chang TC, Chang AC (2025). Antcin K suppresses proinflammatory cytokines expression via the PI3K, Akt and NF-κB pathways in human gingival fibroblasts: implications for periodontitis treatment. Cell death discovery.

[34] West NP, Horn PL, Pyne DB, Gebski VJ, Lahtinen SJ, Fricker PA (2014). Probiotic supplementation for respiratory and gastrointestinal illness symptoms in healthy physically active individuals. Clinical Nutrition.

[35] Vonaesch P, Garneau JR, Dominguez-Bello MG (2025). From global to local: rethinking the design of probiotic intervention strategies. Trends in microbiology.

[36] Rahman SO, Bariguian F, Mobasheri A (2023). The Potential Role of Probiotics in the Management of Osteoarthritis Pain: Current Status and Future Prospects. Curr Rheumatol Rep.

[37] Sophocleous A, Azfer A, Huesa C, Stylianou E, Ralston SH (2023). Probiotics Inhibit Cartilage Damage and Progression of Osteoarthritis in Mice. Calcif Tissue Int.

[38] Zheng H, Chen C (2015). Body mass index and risk of knee osteoarthritis: systematic review and meta-analysis of prospective studies. BMJ Open.

[39] Zhang HQ, Ding TT, Zhao JS, Yang X, Zhang HX, Zhang JJ (2009). Therapeutic effects of Clostridium butyricum on experimental colitis induced by oxazolone in rats. World J Gastroenterol.

[40] Wieland HA, Michaelis M, Kirschbaum BJ, Rudolphi KA (2005). Osteoarthritis - an untreatable disease?. Nature reviews Drug discovery.

[41] Chang TK, Ho TL, Lin YY, Thuong LHH, Lai KY, Tsai CH (2025). Ugonin P facilitates chondrogenic properties in chondrocytes by inhibiting miR-3074-5p production: implications for the treatment of arthritic disorders. International journal of biological sciences.

[42] Lee KT, Chen BC, Liu SC, Lin YY, Tsai CH, Ko CY (2021). Nesfatin-1 facilitates IL-1beta production in osteoarthritis synovial fibroblasts by suppressing miR-204-5p synthesis through the AP-1 and NF-kappaB pathways. Aging (Albany NY).

[43] Palukuru UP, McGoverin CM, Pleshko N (2014). Assessment of hyaline cartilage matrix composition using near infrared spectroscopy. Matrix Biol.

[44] Zhang Z (2015). Chondrons and the pericellular matrix of chondrocytes. Tissue Eng Part B Rev.

[45] Krishnan Y, Grodzinsky AJ (2018). Cartilage diseases. Matrix Biol.

[46] Liu SC, Tsai CH, Wu TY, Tsai CH, Tsai FJ, Chung JG (2019). Soya-cerebroside reduces IL-1β-induced MMP-1 production in chondrocytes and inhibits cartilage degradation: implications for the treatment of osteoarthritis. Food Agr Immunol.

